# Loss of Jak2 Selectively Suppresses DC-Mediated Innate Immune Response and Protects Mice from Lethal Dose of LPS-Induced Septic Shock

**DOI:** 10.1371/journal.pone.0009593

**Published:** 2010-03-09

**Authors:** Jixin Zhong, Ping Yang, Kenjiro Muta, Robert Dong, Mario Marrero, Feili Gong, Cong-Yi Wang

**Affiliations:** 1 Department of Immunology, Tongji Medical College, Huazhong University of Science and Technology, Wuhan, Hubei, China; 2 Center for Biotechnology and Genomic Medicine, Medical College of Georgia, Augusta, Georgia, United States of America; 3 Center for Biomedical Research, Tongji Hospital, Huazhong University of Science and Technology, Wuhan, Hubei, China; 4 Vascular Biology Center, Medical College of Georgia, Augusta, Georgia, United States of America; The University of Chicago, United States of America

## Abstract

Given the importance of Jak2 in cell signaling, a critical role for Jak2 in immune cells especially dendritic cells (DCs) has long been proposed. The exact function for Jak2 in DCs, however, remained poorly understood as Jak2 deficiency leads to embryonic lethality. Here we established Jak2 deficiency in adult *Cre^+/+^Jak2^fl/fl^* mice by tamoxifen induction. Loss of Jak2 significantly impaired DC development as manifested by reduced BMDC yield, smaller spleen size and reduced percentage of DCs in total splenocytes. Jak2 was also crucial for the capacity of DCs to mediate innate immune response. *Jak2^−/−^* DCs were less potent in response to inflammatory stimuli and showed reduced capacity to secrete proinflammatory cytokines such as TNFα and IL-12. As a result, *Jak2^−/−^* mice were defective for the early clearance of *Listeria* after infection. However, their potency to mediate adaptive immune response was not affected. Unlike DCs, *Jak2^−/−^* macrophages showed similar capacity secretion of proinflammatory cytokines, suggesting that Jak2 selectively modulates innate immune response in a DC-dependent manner. Consistent with these results, *Jak2^−/−^* mice were remarkably resistant to lethal dose of LPS-induced septic shock, a deadly sepsis characterized by the excessive innate immune response, and adoptive transfer of normal DCs restored their susceptibility to LPS-induced septic shock. Mechanistic studies revealed that Jak2/SATA5 signaling is pivotal for DC development and maturation, while the capacity for DCs secretion of proinflammatory cytokines is regulated by both Jak2/STAT5 and Jak2/STAT6 signaling.

## Introduction

Dendritic cells (DCs) are the most potent antigen presenting cells (APCs) known today [1]. Other than their well-recognized role in mediating adaptive immune response, they also serve as a key component of innate immunity and bridge innate and adaptive immune response to bacteria and other pathogens [2]–[4]. The pathogen associated molecular patterns (PAMPs) or damage associated molecular patterns (DAMPs) are first sensed by pathogen recognition receptors (PRRs) expressed on their surface, followed by initiating a serial prompt responses such as endocytosis and cytokine secretion. Immune disorders are a system out of balance as manifested by either excessive or defective response, and septic shock is a typical example caused by the excessive innate immune response [5]–[10]. Septic shock (or endotoxic shock) is a severe sepsis with organ hypoperfusion and hypotension that are poorly responsive to initial fluid resuscitation. The mortality rate in patients with septic shock ranges from 20 to 80%, and in the USA alone it is estimated that more than 100,000 deaths occur each year [11], [12]. Therefore, septic shock has been accounted for the most common cause of death in the intensive care unit [13]–[15]. Given the importance of DCs in the vanguard of innate immune response, research into the development of new septic therapeutics has focused more and more on their crucial role in orchestrating the initial host response to infection [16], but the advancement has been painfully slow and fraught with difficulties. The ideal therapeutic target for septic shock would be directed to selectively modulate innate immune response without affecting adaptive defense. Nevertheless, it would be a formidable challenge to characterize such a target among all immune regulatory molecules within the genome.

Jak2 is one of the four janus kinase members identified in mammals [17], [18]. It acts as a critical component of signal pathways involved in cellular survival, proliferation, differentiation and apoptosis [19]–[21]. Particularly, Jak2 has been suggested to be crucial for the regulation of DC development and functionality [22]–[25]. However, the exact function for Jak2 in DCs remained, somehow, controversial depending on each particular chemical inhibitor used. In the present study, we induced Jak2 deficiency in adult mice by crossing *Jak2^fl/fl^* mice with Cre-ERT2 transgenic mice. Loss of Jak2 only selectively suppresses the capacity of DCs to initiate innate immune response, but there is no discernable impact on their capacity to mediate adaptive immune response. As a result, *Jak2^−/−^* mice are remarkably resistant to lethal dose of LPS-induced septic shock. Our results suggest that Jak2 could be a unique therapeutic target for the intervention and treatment of clinical septic shock.

## Results

### Jak2 Is Essential for DC Development and Maturation

**Figure 1:**
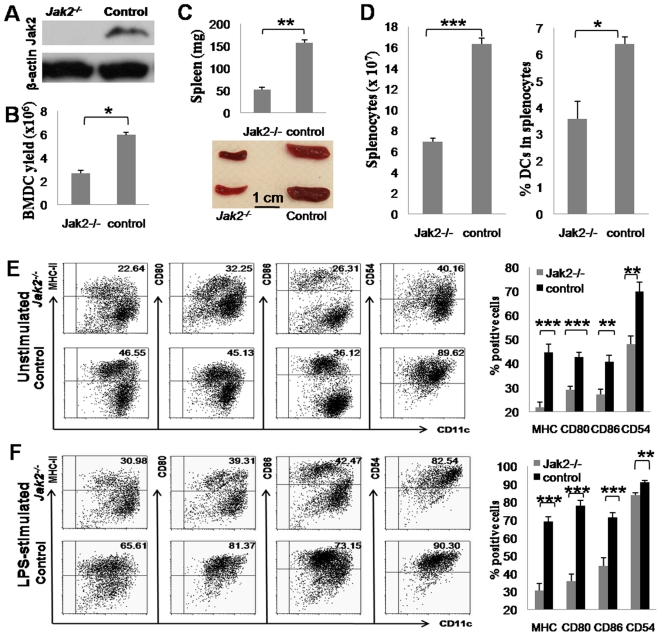
Jak2 is essential for DC development and maturation. (A) Tamoxifen efficiently induced Jak2 deficiency in DCs. BMDCs were generated from *Cre^+/+^Jak2^fl/fl^* mice after 2 wk of tamoxifen or carrier solution induction and then subjected to Western blot analysis of Jak2 expression. High levels of Jak2 were detected in control mice, while Jak2 was absent in tamoxifen induced mice. (B) Loss of Jak2 significantly reduced BMDC yield. Bone marrow cells (1×10^7^) were used to generate BMDCs using GM-CSF (10 ng/ml) and IL-4 (5 ng/ml) as described. Bone marrow cells deficient for Jak2 showed 1.3-fold decrease of BMDC yield. (C) *Jak2^−/−^* mice show significant smaller spleen size. Top panel: a bar graphic figure showing the average spleen weight of all mice analyzed; bottom panel: representative spleens from *Jak2^−/−^* and control mice. (D) Analysis of splenocytes in *Jak2^−/−^* and control mice. Both total splenocytes and the percentage of splenic DCs in total splenocytes were reduced in *Jak2^−/−^* mice as compared with that of controls. (E) BMDC surface marker expressions in the unstimulated condition. Left panel: a representative of flow cytometry data; right panel: a bar graphic figure showing the average percentage of BMDCs positive for each surface marker analyzed. (F) BMDC surface marker expressions after LPS stimulation. Similar as above, left panel is a representative of flow cytometry data, and right panel shows the average percentage of BMDCs positive for the corresponding surface marker. All data are present as mean ± SE of four replicates. *, *p*<0.05; **, *p*<0.01; ***, *p*<0.001.

*Cre^+/+^Jak2^fl/fl^* mice were generated by crossing Cre-ERT2 transgenic mice with *Jak2^fl/fl^* mice as described. For induction of Jak2 deficiency, 8 wk-old male *Cre^+/+^Jak2^fl/fl^* mice were i.p. injected with tamoxifen (25 mg/kg body weight) for five consecutive days. Male littermates administered with carrier solution (10% ethanol in corn oil) were used as controls. To confirm Jak2 deficiency, the mice were sacrificed after 2 wk of last injection. Bone marrow derived dendritic cells (BMDCs) and splenocytes were prepared and subjected to Western blot analysis of Jak2 expression. As shown in Figure 1A, BMDCs originated from control mice showed high levels of Jak2 expression, while Jak2 was undetectable in BMDCs of tamoxifen induced mice. Similar results were also observed in splenocytes (data not shown). Collectively, these results indicate that tamoxifen efficiently induced Jak2 deficiency in *Cre^+/+^Jak2^fl/fl^* mice.

We first sought to address the impact of Jak2 deficiency on DC development. To this end, 1×10^7^ bone marrow cells originated from *Jak2^−/−^* and control littermates were induced with GM-CSF and IL-4 to generate BMDCs, respectively. Jak2 deficiency significantly reduced DC production, a 1.3-fold decrease for BMDC yield was observed in *Jak2^−/−^* mice as compared with that of control mice (Figure 1B). We also noticed a significant smaller size for spleens in *Jak2^−/−^* mice than that of control mice (52±8 mg vs. 157±10 mg, *p*<0.001; Figure 1C). In line with this observation, total splenocytes in *Jak2^−/−^* mice were significantly less than that of controls (Figure 1D, left panel). Next, we examined splenic DCs. To our surprise, in addition to the reduced number of total splenocytes, the percentage of DCs in spenocytes has also significantly decreased (Figure 1D, right panel). In contrast, we failed to detect a significant alteration for the percentage of CD4 and CD8 T cells in total spenocytes (data not shown). Together, these data suggest that loss of Jak2 significantly impaired DC development.

We next examined DC phenotypic differences between *Jak2^−/−^* and control mice. For this purpose, day-9 BMDC cultures were stimulated with 0.5 µg/ml LPS overnight and harvested on day-10 for flow cytometry analysis of surface marker expressions. Both *Jak2^−/−^* and control bone marrow cells produced >85% purity of DCs, which were then gated for the analysis of surface MHC-II, CD80, CD86, and CD54 expressions. In contrast to previous published data [26], [27], loss of Jak2 rendered DCs significant less potent in response to maturation stimulation. We first noticed that much lower percentage of *Jak2^−/−^* BMDCs expressing MHC-II and co-stimulatory molecules such as CD80, CD86 and CD54 before stimulation (Figure 1E). Upon LPS stimulation, majority of control BMDCs became matured as characterized by high levels of MHC-II, CD80, CD86 and CD54 expressions. In sharp contrast, only a small proportion of *Jak2^−/−^* BMDCs became matured (Figure 1F). To further confirm this observation, we analyzed splenic DCs. Single splenic cells were prepared and then co-stained for CD11c and MHC-II or one of the above indicated costimulatory molecules. Similar as BMDCs, significant lower percentage of *Jak2^−/−^* splenic DCs expressed high levels of MHC-II and costimulatory molecules (Figure S1). To further confirm these observations, we treated BMDCs with AG490, a broadly used Jak2 inhibitor. In contrast to previously published data [27], but consistent with our current data observed on *Jak2^−/−^* mice, AG490 significantly suppressed DC maturation (Figure S2). We also tested the impact of tamoxifen on DC maturation and failed to detect any perceptible effect (Figure S3).

We next examined the impact of Jak2 deficiency on macrophages, another important type of professional APCs. Peritoneal exudate macrophages (PEM) were collected by peritoneal lavage as described. Western blot analysis confirmed the absence of Jak2 in PEM derived from *Jak2^−/−^* mice (data not shown). The cells were then subjected to flow cytometry analysis as above. To our surprise, we failed to observe a significant difference for the number of macrophages between *Jak2^−/−^* and control mice, and Western analysis also confirmed Jak2 deficiency in PEM derived from *Jak2^−/−^* mice (data not shown). However, similar as DCs, *Jak2^−/−^* macrophages showed a less matured phenotype characterized by the lower percentage of cells expressing MHC-II and costimulatory molecules as compared with that of control mice (Figure S4).

### Jak2 Deficiency Attenuates the Capacity of DCs to Initiate Innate Immune Response

**Figure 2:**
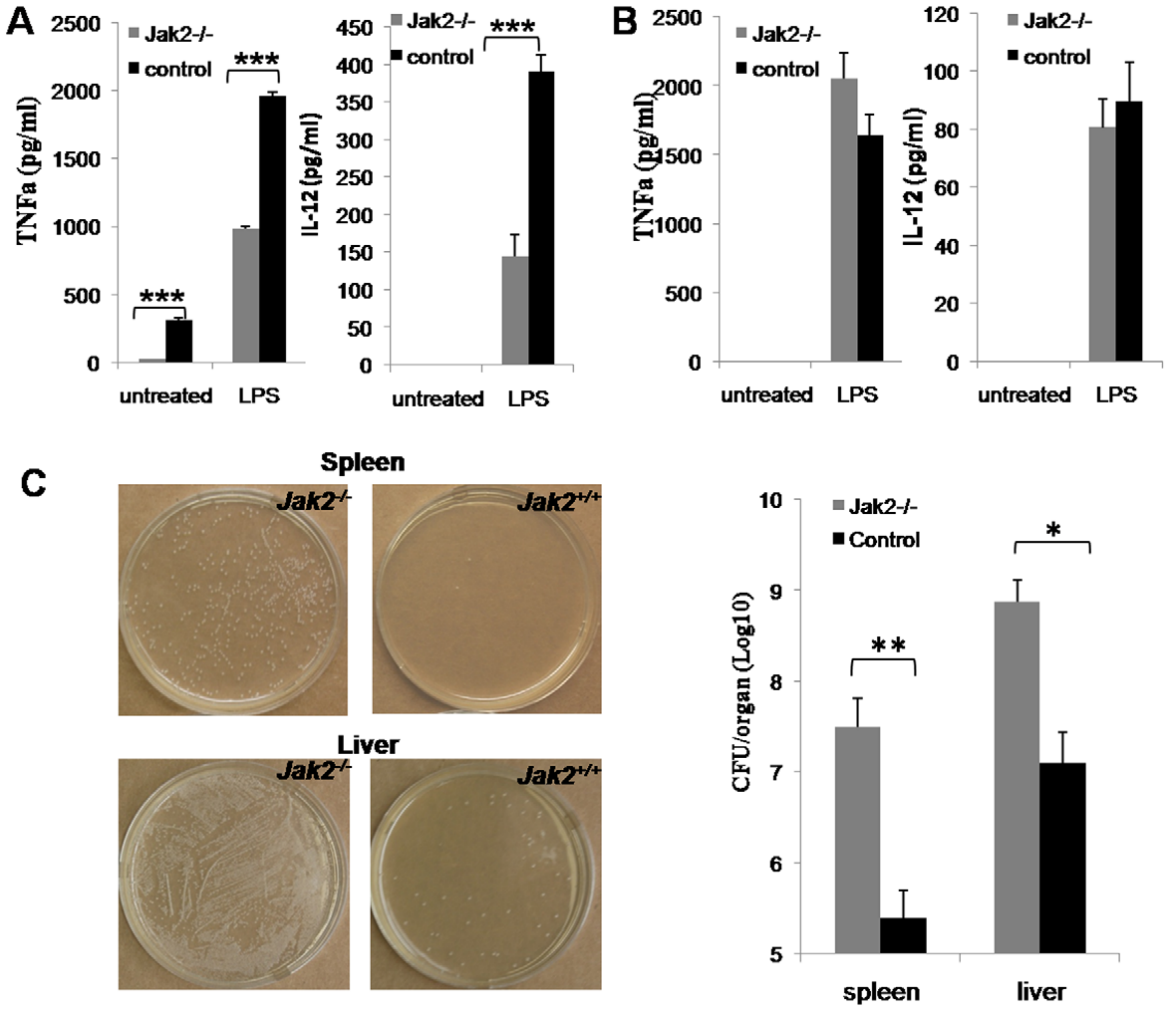
Jak2 deficiency selectively attenuates the capacity of DCs to initiate innate immune response. (A) Loss of Jak2 attenuates the capacity of DCs secretion of proinflammatory cytokines. Supernatants of BMDCs with or without LPS stimulation were harvested and subjected to ELISA analysis of cytokine secretion. BMDCs deficient for Jak2 showed significant reduced capacity to secrete TNFα and IL-12. (B) Jak2 failed to show perceptible impact on macrophages secretion of proinflammatory cytokines. PEM derived from *Jak2^−/−^* and control mice were cultured with or without LPS stimulation and supernatants were harvested for ELISA analysis as above, respectively. (C) *Jak2^−/−^* mice showed impaired early clearance of *Listeria*. *Jak2^−/−^* and control mice were i.v. injected with ∼1 LD50 *Listeria Monocytogenes* in PBS. Spleens and livers were harvested after 48 h infection. Serial 10-fold dilutions of organ homogenates were plated on TSB-agar and CFU were counted after 48 h incubation. Left panel: a representative results for cultures inoculated with homogenates; right panel: a bar graphic figure showing the average CFU/organ of 3 independent experiments. All data are present as mean ± SE. *, *p*<0.05; **, *p*<0.01; ***, *p*<0.001.

Next, we examined the capacity of *Jak2^−/−^* DCs secretion of proinflammatory cytokines such as TNFα and IL-12 using the above culture supernatants. Before stimulation IL-12 was undetectable in both wild-type and *Jak2^−/−^* cultures, while low levels of TNFα were detected, but the amount in *Jak2^−/−^* cultures was 12-fold lower than that of control cultures (Figure 2A). Upon LPS stimulation BMDCs secreted copious amounts of TNFα and IL-12, and consistently, *Jak2^−/−^* BMDCs secreted much lower levels of TNFα and IL-12 as compared with that of control BMDCs (Figure 2A). However, both wild-type and *Jak2^−/−^* BMDCs showed similar capacity for secretion of IL-10 (Figure S5A).Similarly, we analyzed cytokine secretion from *Jak2^−/−^* macrophages. Supernatants harvested from above PEM cultures were examined for TNFα and IL-12 production by ELISA assay. Very surprisingly, unlike *Jak2^−/−^* DCs, macrophages deficient for Jak2 showed similar capacity to secrete TNFα and IL-12 as that of wild-type cells (Figure 2B). All together, these data suggest that loss of Jak2 only selectively impairs the capacity of DCs to initiate innate immune response.

To further confirm above observations, we checked *Listeria* clearance in *Jak2^−/−^* mice as innate immunity especially the production of proinflammatory cytokines plays a pivotal role in the early clearance of *Listeria* after infection [28]–[31]. For this purpose, Mice were systemically infected by i.v. injection with 1×10^6^ CFU (∼1 LD_50_) of *Listeria*, and organs from *Jak2^−/−^* and control mice were harvested after 2-day of infection. Dilutions of spleen and liver homogenates were plated on TSB-agar and colonies were counted after 2-day of culture, respectively. In line with above results, *Jak2^−/−^* mice showed significant lower capacity for early clearance of *Listeria* as manifested by 50–100 times more colonies observed in *Jak2^−/−^* mice as compared with that of control mice (Figure 2C).

### *Jak2^−/−^* DCs Possess Similar Capacity to Mediate Adaptive Immune Response

**Figure 3:**
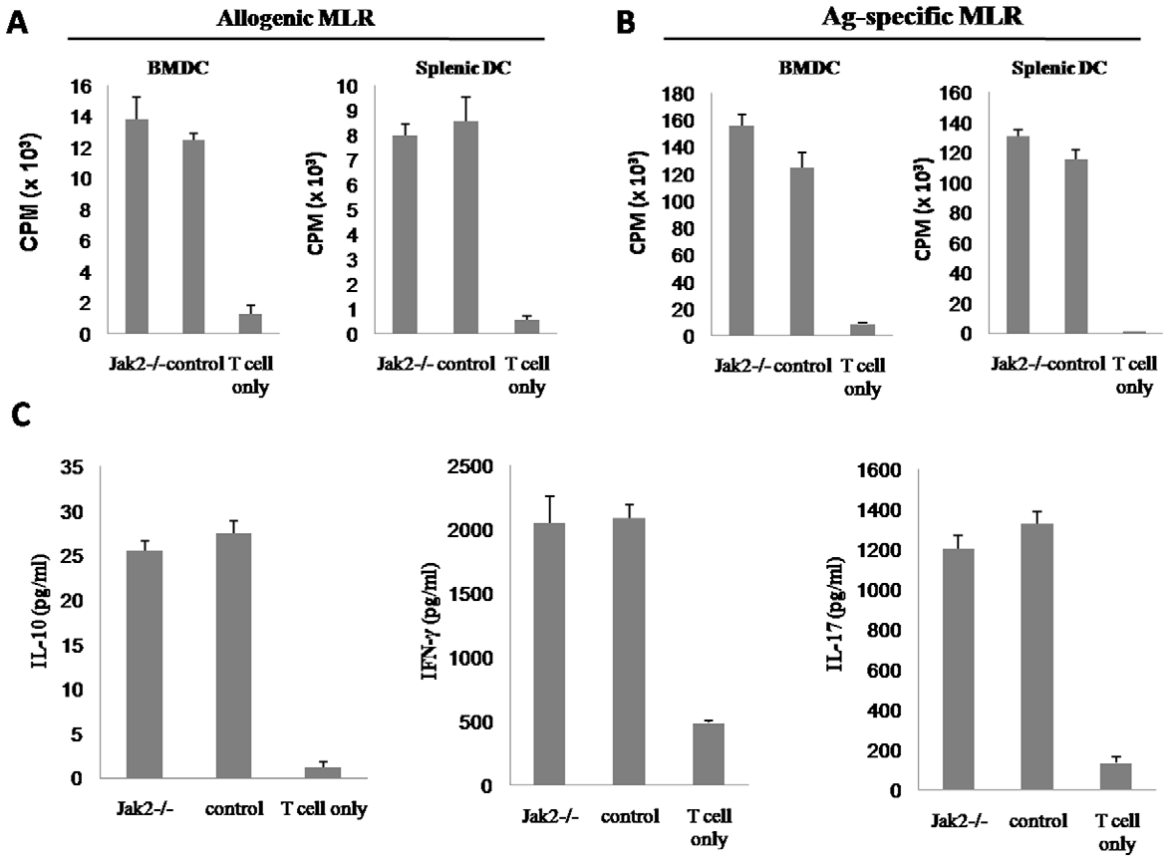
DCs deficient for Jak2 show similar potency to stimulate T cell activation. (A) Allogenic MLR results. (B) Antigen-specific MLR results. (C) ELISA analysis of cytokines produced by the activated T cells. MLR were carried out using either BALB/c T cells (allogenic) or OT-1 T cells (antigen-specific) as responders in the condition of DC/T ratio 1∶10. T cell proliferation was estimated by ^3^H thymidine incorporation. Data shown in the figure are mean ± SE of three independent experiments performed.

The above results prompted us to check whether Jak2 deficiency would affect the capability of DCs to mediate adaptive immune response. We first performed allogenic MLR to examine the capacity of DCs to stimulate T cell proliferation. To this end, irradiated *Jak2^−/−^* and control BMDCs were cocultured with T cells originated from BALB/c mice, and T cell proliferation was determined by ^3^H thymidine incorporation. Unexpectedly, *Jak2^−/−^* BMDCs showed similar potency as control BMDCs to stimulate alloreactive BALB/c T cell proliferation (Figure 3A, left panel). To confirm this observation, we performed similar studies using splenic DCs. Splenocytes pooled from 5 *Jak2^−/−^* or control mice were used to enrich splenic DCs as described. Irradiated splenic DCs (2000 rads) were then cocultured with BALB/c T cells as above, respectively. Consistently, splenic *Jak2^−/−^* DCs were potent to stimulate alloreactive T cell activation (Figure 3A, right panel). We next examined the capacity of *Jak2^−/−^* DCs to initiate antigen-specific T cell activation. For this purpose, irradiated *Jak2^−/−^* BMDCs or splenic DCs were first pulsed with 1 µM OVA peptide, and then cocultured with OT-1 T cells as above. Similarly, both *Jak2^−/−^* BMDCs and splenic DCs were potent to stimulate antigen-specific T cell activation (Figure 3B).

Next, we analyzed cytokine profile of T cells activated by *Jak2^−/−^* DCs. We selectively analyzed the production of IFN-γ (Th1), IL-10 (Th2) and IL-17 (Th17) by ELISA analysis of above collected culture supernatants. Once again, *Jak2^−/−^* DCs were as potent as control DCs to stimulate T cells secretion of IFN-γ, IL-10, and IL-17 (Figure 3C). Taken together, these results indicate that loss of Jak2 only selectively inhibits the potency of DCs to initiate innate immune response without perceptible effect on DC-mediated adaptive immune response.

### *Jak2^−/−^* Mice Are Remarkably Resistant to Lethal Dose of LPS-Induced Septic Shock

**Figure 4:**
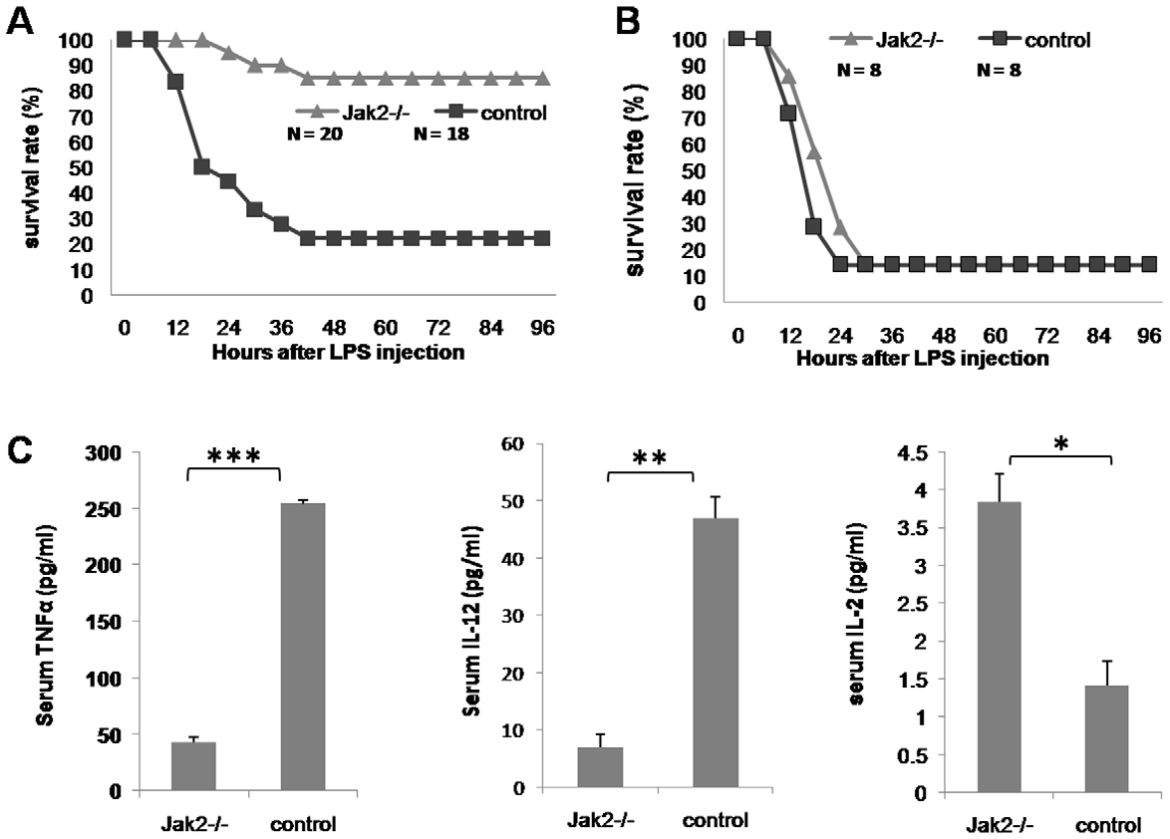
*Jak2^−/−^* mice show DC-dependent resistance to lethal dose of LPS-induced septic shock. (A) The survival rates of *Jak2^−/−^* and control mice were monitored for 4 days after lethal dose of LPS challenge. It was noticed that mice would completely recover from septic shock if they did not die within 36 h of lethal dose of LPS challenge. 85% of *Jak2^−/−^* mice (17 out of 20) have completely recovered from septic shock, while only 22% of control mice (4 out of 18) have survived (*p*<0.0001). (B) Adoptive transfer of normal DCs into irradiated *Jak2^−/−^* mice restored their susceptibility to LPS-induced septic shock. Irradiated *Jak2^−/−^* or control mice were first adoptively transferred with 1×10^7^ normal BMDCs followed by lethal dose of LPS challenge. *Jak2*−/− mice after adoptive transfer of normal DCs showed similar survival rate as that of control mice, indicating that the resistant phenotype for septic shock seen in *Jak2^−/−^* mice is DC-dependent. (C) ELISA analysis of serum cytokines of nonlethal dose of LPS challenged mice. Both *Jak2^−/−^* and control mice were first challenged with nonlethal dose of LPS (150 µg/mouse) and 12 h later the mice were sacrificed, respectively. Sera were pooled from three mice and then subjected to ELISA analysis of cytokine production. Three pools were analyzed for each study group. *Jak2^−/−^* mice produced significant lower levels of proinflammatory cytokines as compared with that of control mice. The data are present as mean ± SE of three replicates. *, *p*<0.05; **, *p*<0.01; ***, *p*<0.001.

Given the role that Jak2 selectively regulates DC-mediated innate immune response, we next examined its implication in the pathogenesis of septic shock, in which excessive innate immune response is suggested to be responsible for the disease etiology [32]–[34]. To this end, 8 wk-old male *Cre^+/+^Jak2^fl/fl^* mice were first induced with tamoxifen or control vehicle as above. Four weeks later, lethal dose of LPS (50 mg/kg body weight) was then administered into each mouse *via* i.p. injection. In general, all control mice were much weaker as compared with that of *Jak2^−/−^* mice after 6–12 h of injection, and remarkably, 14 out of 18 control mice (78%) died within 36 h of injection. In sharp contrast, 17 out of 20 *Jak2^−/−^* mice have completely recovered from lethal dose of LPS challenge (Figure 4A, 85% vs. 22%, *p*<0.0001), indicating that loss of Jak2 protected mice from lethal dose of LPS-induced septic death.

To confirm that the protective effect observed in *Jak2^−/−^* mice was DC-dependent, we next performed an adoptive transfer study using DCs originated from wild-type mice. To this end, the irradiated *Jak2^−/−^* mice and their corresponding controls were adoptively transferred 1×10^7^ normal BMDCs followed by challenging with lethal dose of LPS as described. As expected, both *Jak2^−/−^* and control mice showed similar survival rate after lethal dose of LPS challenge (Figure 4B), demonstrating that DCs with reduced capacity to initiate innate immune response protected *Jak2^−/−^* mice from septic shock.

To further address above question, we examined serum TNFα and IL-12 production in mice challenged with nonlethal dose of LPS (150 µg/mouse). Serum samples were collected from both *Jak2^−/−^* and control mice after 12 h of LPS challenge. Sera pooled from 3 mice of each study group were subjected to ELISA analysis of cytokine production. Consistent with above observations, *Jak2^−/−^* mice produced significant lower levels of serum TNFα and IL-12 as compared with that of control mice (Figure 4C). Interestingly, we observed much higher levels of serum IL-2 in *Jak2^−/−^* mice although IL-2 levels for both *Jak2^−/−^* and control mice were relatively low (Figure 4C). Similar as in vitro studies, we failed to detect significant differences for serum IL-10 between *Jak2^−/−^* and control mice after LPS challenge (Figure S5B). As DC apoptosis is a potential causative factor for post-septic death, we examined DC apoptosis by flow cytometry. BMDCs were treated with 10 µg/ml LPS for 96 h and then stained with Annexin-V and PI for flow cytometry analysis. We failed to detect a significant difference of apoptosis between *Jak2^−/−^* and control BMDCs (Figure S6A). We further analyzed splenic DC apoptosis in *Jak2^−/−^* mice after 20 h of LPS challenge (150 µg/mouse), and similar results were obtained (Figure S6B).

### Loss of Jak2 Impairs the Activation of STAT3, 4, 5, and 6

**Figure 5:**
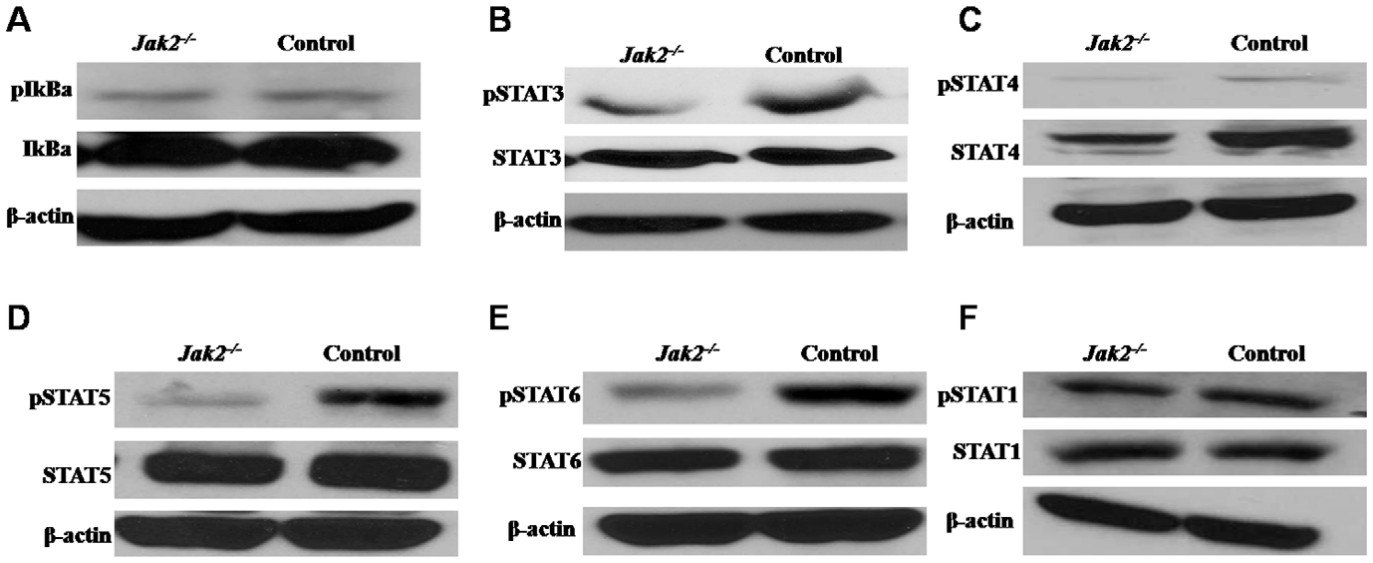
Western blot analysis of the activation states of Jak2 downstream molecules. 2×10^6^/ml *Jak2^−/−^* and control BMDCs were harvested after 30 min of LPS stimulation (1 µg LPS/ml) and cell lysates were prepared for Western blot analysis of the phosphorylated form of signaling molecules. (A) pIκBα; (B) phosphorylated STAT3 (pSTAT3); (C) pSTAT4; (D) pSTAT5; (E) pSTAT6; and (F) pSTAT1. Four independent experiments were performed and consistent results were obtained.

To investigate the underlying mechanisms by which Jak2 regulates DC-mediated innate immune response, 2×10^6^/ml *Jak2^−/−^* or control BMDCs were stimulated with 1 µg/ml LPS for 30 min and then harvested for Western blot analysis of target molecules. Given the importance of NFκB signaling in LPS-induced immune response, we first examined phosphorylated IκBα (pIκBα) in BMDC lysates. To our surprise, we failed to detect a significant difference for pIκBα between *Jak2^−/−^* and control BMDCs (Figure 5A). Therefore, Jak2 regulation of DCs for secretion of proinflammatory cytokines seems to be independent of NFκB signaling. We next examined the activation states of downstream signaling molecules. As STAT signaling has been demonstrated to be crucial for cytokine receptors to transduce intracellular signals, we therefore checked the phosphorylation states of transcription factors STAT1, STAT3, STAT4, STAT5 and STAT6. It was found that loss of Jak2 significantly attenuated STAT3, 4, 5 and 6 activation in BMDCs as manifested by the significant decreased levels for phosphorylated STAT3 (pSTAT3, Figure 5B), pSTAT4 (Figure 5C), pSTAT5 (Figure 5D) and pSTAT6 (Figure 5E). In contrast, Jak2 did not show a perceptible effect on STAT1 activation (Figure 5F). To demonstrate why macrophages deficient for Jak2 showed similar capacity secretion of proinflammatory cytokines, we further examined the activation states of all STAT factors in *Jak2^−/−^* macrophages as above. Unlike DCs, loss of Jak2 in macrophages only affected the activation of STAT3 and 4 (Figure S7).

### Jak2/STAT5 Signaling Is Indispensable for DC Development and Maturation

**Figure 6:**
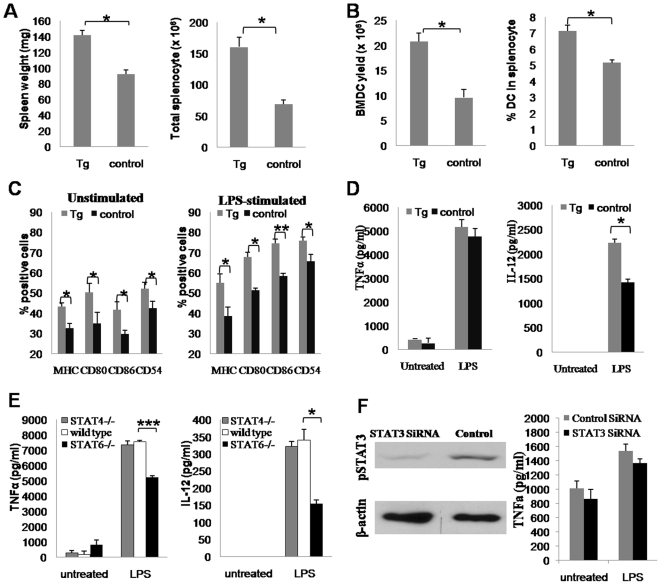
Jak2/STAT5 signaling is indispensable for DC development and maturation, while the capacity of DC for secretion of proinflammatory cytokines is regulated by both Jak2/STAT5 and Jak2/STAT6 signaling. (A) Stat5-Tg mice show larger size for the spleen and higher number of splenocytes. (B) Transgenic *Stat5* expression enhances BMDC yield and increases the percentage of splenic DCs in total splenocytes (C) DCs with transgenic *Stat5* expression show higher potency in response to LPS stimulation. Significant higher percentage of DCs show matured phenotype as characterized by high levels of MHC II and costimulatory molecule expressions before/after LPS stimulation. (D) ELISA analysis of cytokines secreted by DCs with transgenic *Stat5* expression. Stat5-Tg BMDCs showed similar capacity for secretion of TNFα, while the production of IL-12 was significantly higher than that of control BMDCs after LPS stimulation. (E) ELISA analysis of cytokine secretion by BMDCs generated from *Stat4^−/−^* and *Stat6^−/−^* mice. *Stat6^−/−^* BMDCs showed significant lower capacity for secretion of TNFα and IL-12 upon LPS stimulation. In contrast, *Stat4^−/−^* BMDCs showed similar capacity for secretion of above indicated cytokines as control BMDCs. (F) Down-regulation of *Stat3* in DC2.4 cell by siRNA. DC2.4 cells were first transfected with a *Stat3*-specific siRNA or a scramble RNA followed by LPS stimulation for analysis of cytokine secretion. Left panel: Western blot results showing significant down-regulation of pSTAT3 in cells transfected with siRNA. Right panel: ELISA analysis of cytokine secretion. IL-12 was undetectable in both siRNA and scramble transfected DC2.4 cells, while siRNA transfected DC2.4 cells showed similar capacity for secretion of TNFα. Data shown in the figure are presented as mean ± SE. *, *p*<0.05; **, *p*<0.01; ***, *p*<0.001.

We next sought to examine the functional relevance of Jak2/STAT5 signaling in DCs. Stat5-Tg mice and their control littermates were used for the study. In contrast to *Jak2^−/−^* mice, the weight of spleens and number of splenocytes were significantly higher in Stat5-Tg mice as compared with their control littermates (Figure 6A). Consistent with this observation, the yield of BMDCs from Stat5-Tg mice was 1.6-fold higher than that of their control littermates (Figure 6B, left panel). Similarly, the percentage of splenic DCs in total splenocytes was significantly higher than their control littermates (Figure 6B, right panel). We further examined the impact of Jak2/STAT5 signaling on DC maturation. Similar as above, DCs were generated from bone marrow cells originated from Stat5-Tg mice and control littermates, respectively, and then stimulated with LPS overnight. As shown in Figure 6C, significant higher percentage of BMDCs derived from Stat5-Tg mice expressing high levels of MHC-II and costimulatory molecules such as CD80, CD86 and CD54 before and after LPS stimulation, as compared with their control littermates. We next examined cytokine secretion using the above culture supernatants. Interestingly, BMDCs with transgenic *Stat5* expression showed similar capacity to secret TNFα, however, much higher levels of IL-12 was noted (Figure 6D). Together, our results suggest that Jak2/STAT5 signaling plays an indispensable role in DC development, maturation and IL-12 secretion.

### Jak2/STAT6 Is Essential for DCs Secretion of Proinflammatory Cytokines

To delineate whether Jak2/STAT4 and Jak2/STAT6 signaling implicate in DC development and DC-mediated innate immune response, *Stat4^−/−^* and *Stat6^−/−^* mice were used for the study. It is noteworthy that we failed to detect significant alterations for DC development and maturation in both *Stat4^−/−^* and *Stat6^−/−^* mice (data not shown). As a result, our next focus is to examine their implications in the regulation of cytokine secretion. Before stimulation, BMDCs only secreted low levels of TNFα, while IL-12 was undetectable in the cultures of all BMDCs (Figure 6E). However, high levels of TNFα and IL-12 were detected after LPS stimulation and *Stat6^−/−^* BMDCs showed significant lower capacity to secret TNFα and IL-12, although we observed relatively higher levels of TNFα in *Stat6^−/−^* BMDCs in the unstimulated condition (Figure 6E). All of these results demonstrate that Jak2/STAT6 signaling regulates the capacity of DCs to secrete cytokines implicated in innate immune response. To our surprise, we failed to detect a significant impact for Jak2/STAT4 signaling on either DC development or cytokine secretion. Previous studies demonstrated that STAT4 levels directly correlate with IL-12-dependent IFNγ production by DCs during antigen presentation [35], it is possible that Jak2/STAT4 signaling implicates in DC-mediated T cell polarization. Consistent with this assumption, we failed to detect DCs originated from both *Jak2^−/−^* and control mice secretion of IFNγ after LPS stimulation despite lower percentage of DCs carrying high levels of MHC-II and costimulatory molecules in *Jak2^−/−^* mice (data not shown).

As mice deficient for *Stat3* lead to embryonic lethality [36], we used DC2.4 cells, a B6-derived DC line, for the study. DC2.4 cells were transfected with a *Stat3* siRNA or a scramble RNA as described. Western blot analysis confirmed a significant reduction for the active form of STAT3 in response to LPS stimulation in cells transfected with siRNA (Figure 6F, left panel). Culture supernatants were then collected and subjected to analysis of proinflammatory cytokine secretion. To our surprise, we failed to detect IL-12 production in all cultures either before or after LPS stimulation. However, DC2.4 cells secreted high levels of TNFα, but no significant difference was noted between cells transfected with siRNA and scramble RNA (Figure 6F, right panel), indicating that Jak2/STAT3 signaling may not affect the capacity of DCs secretion of proinflammatory cytokines. Several recent studies suggested a role for *Stat3* in DC development and differentiation [37]–[39], and therefore, Jak2/STAT3 is possibly to work together with Jak2/STAT5 signaling involved in DC development.

## Discussion

Given the importance of Jak2 in both physiological and pathological conditions, a critical role for Jak2 in immune cells especially DCs has long been proposed. However, despite past extensive studies, its exact function in DCs remains controversial [26], [27], [35], [40]. The main reason for the discrepant results is that most studies were conducted using Jak2 inhibitors as loss of Jak2 is embryonic lethal, while those inhibitors could non-specifically target other molecules. In this study, we have generated an inducible Jak2 deficient model by crossing *Jak2^fl/fl^* mice with Cre-ERT2 transgenic mice. We first performed studies to examine the implication of Jak2 in DC development. We have demonstrated that loss of Jak2 impairs DC development as manifested by the reduced BMDC yield, smaller spleen size, and reduced percentage of DCs in total splenocytes (Figures 1B–D). Other than this recognized role, we further noticed a pivotal role for Jak2 in the regulation of the capacity for DCs to mediate innate immune response. *Jak2^−/−^* DCs were found to be less potent in response to inflammatory stimuli (Figures 1E & 1F), and furthermore, they showed significant reduced capacity to secrete proinflammatory cytokines such as TNFα and IL-12 upon LPS stimulation (Figure 2A). As a result, *Jak2^−/−^* mice were defective for the early clearance of *Listeria* after infection as compared with that of control mice (Figure 2C). However, their potency to mediate adaptive immune response such as the capacity to activate allogenic or antigen specific T cells was not affected (Figures 3A–C). Very interestingly, it appears that Jak2 regulation of innate immune response is DC-dependent as macrophages deficient for Jak2 showed similar capacity secretion of proinflammatory cytokines such as TNFα and IL-12 (Figure 2B). This discrepancy could be caused by the difference for the STAT activation profile between DCs and macrophages. For example, Jak2 deficiency affected the activation of STAT3, 4, 5 and 6 in DCs (Figure 5), while defective activation was only noted for STAT3 and 4 in *Jak2^−/−^* macrophages (Figure S7). Together, our data suggest that Jak2 selectively modulates innate immune response which appears to be DC-dependent.

To further dissect the role of Jak2 in DC-mediated innate immune response, we used septic shock, a typical disorder characterized by the uncontrolled innate immune response, as a model for the study. Septic shock is a severe sepsis due to excessive release of proinflammatory cytokines such as TNFα and IL-12, which then lead to vasodilation, increased vascular permeability, hypotension, multiple organ failure and ultimately shock and death. Unlike septic death resulted from late phase of sepsis, septic shock is manifested by excessive innate immune response such as copious amount of proinflammatory cytokine release. Therefore, Jak2 signaling might be a potential therapeutic target for controlling excessive innate immune response during septic shock. Cecal ligation and puncture (CLP) is a widely used experimental model for sepsis [41]. However, animals die after CLP usually ranging from 24 h to 7 days which implicates both altered innate immune response and maladaptive immune response. Since our focus is to determine Jak2 deficiency in DC-mediated innate immune response, it would be crucial to ultimately exclude the implication of adaptive immune response. As a result, we have chosen lethal dose of LPS-induced septic shock for the study. As expected, 78% of control mice died from septic shock within 36 h of lethal dose of LPS challenge, while 85% of *Jak2^−/−^* mice have survived from the same dose of LPS challenge (Figure 4A), indicating that *Jak2^−/−^* mice were protected from LPS-induced septic shock. Adoptive transfer studies were then applied to further address that this protective effect is DC-dependent. Since macrophages deficient for Jak2 only showed defective activation for STAT3 and 4 (Figure S7), we selected adoptive transfer of DCs for the study, in which 1×10^7^ normal DCs were transferred into irradiated *Jak2^−/−^* mice. As expected, *Jak2^−/−^* mice after adoptive transfer of normal DCs showed restored susceptibility to LPS-induced septic shock (Figure 4B). These results demonstrate that DCs with reduced capacity for initiation of innate immune response rendered *Jak2^−/−^* mice more resistant to LPS-induced septic shock. In consistent with this conclusion, ELISA analysis of serum cytokines revealed significant lower levels of proinflammatory cytokines such as TNFα and IL-12 in *Jak2^−/−^* mice after 12 h of nonlethal dose of LPS challenge (Figure 4C), while the production of IL-10 was similar between *Jak2^−/−^* and control mice (Figure S5).

Of note, unlike TNFα and IL-12, we noticed much higher levels of serum IL-2 in *Jak2^−/−^* mice after LPS challenge as compared with control mice, although the absolute IL-2 level was relatively low in both strains of mice (Figure 4C). Recent studies demonstrated that microbial stimuli (not proinflammatory cytokines) are able to induce IL-2 secretion by DCs [42]–[44], while we failed to detect IL-2 secretion in DCs following LPS stimulation which could be due to the differences of our culture system or lower sensitivity of our detection system. Given the important regulatory role exerted by IL-2 in the immune system, it has been suggested to be tightly regulated by the STAT5-dependent negative feedback signal [45]. Therefore, the reduced *Stat5* activity probably contributes to the observed higher levels of serum IL-2 in *Jak2^−/−^* mice. Studies have shown that DC-derived IL-2 plays a pivotal role in their capability to prime alloreactive T cells [42], [46], and therefore, enhanced IL-2 production in *Jak2^−/−^* DCs probably contributes to their unchanged potency to stimulate allogenic or antigen specific T cell activation as well.

Given the important role of Jak2 in cell signaling, we anticipate that Jak2 deficiency would affect the activation of multiple signaling pathways of DCs. Unexpectedly, we only detected altered activity for Stat3, 4, 5 and 6 (Figure 5). It seems that loss of Jak2 in DCs has negligible impact on Stat1 and NFκB signaling, which could be due to the compensated effect from other redundant Jak kinases. For example, other than Jak2, Stat1 can be activated by either Jak1 or Tyk2 [47]. Using Stat5-Tg mice, we have demonstrated evidence indicating that Jak2/STAT5 is indispensable for DC development and maturation in response to inflammatory stimulation. In opposite to *Jak2^−/−^* mice, Stat5-Tg mice displayed higher DC yield, larger spleen size and higher potency in response to LPS stimulation (Figure 6). However, Jak2/STAT5 only partly regulates DCs secretion of IL-12, indicating additional signaling pathway(s) that are also implicated in the regulation of DCs secretion of proinflammatory cytokines.

To examine the possible implication of additional signaling pathway(s), we performed studies in *Stat6^−/−^* mice and demonstrated a pivotal role for Jak2/STAT6 signaling in the regulation of cytokine secretion in DCs. We noticed a significant impaired production of TNFα and IL-12 in *Stat6^−/−^* DCs following LPS stimulation, although *Stat6^−/−^* DCs secreted relatively higher baseline of TNFα (Figure 6E). Based on the results for TNFα and IL-12 production, there is no doubt that Jak2/STAT6 signaling regulates the capacity of DCs secretion of proinflammatory cytokines. We further performed similar studies in *Stat4^−/−^* mice and DC2.4 cells transfected with a *Stat3* siRNA. Surprisingly, we failed to detect any perceptible impact for STAT3 and STAT4 on the capacity of DCs for cytokine secretion (Figures 6E & F). Given the importance of STAT4 pathway in T cell polarization [48]–[51], Jak2/STAT4 signaling could be implicated in the regulation of DCs to mediate T cell polarization. On the other hand, STAT3 has been recently suggested to be important for DC development and differentiation [37]–[39], and therefore, Jak2/STAT3 could work together with Jak2/STAT5 signaling to implicate in DC development.

Extensive studies have been conducted for decades to demonstrate the function of Jak2 in immune cells including DCs by using different Jak2 inhibitors. Results derived from these studies, however, remained controversial, because of non-specificity of each Jak2 inhibitor. To our knowledge, the present report is the first study carried out in adult mice deficient for Jak2. Due to the complexity of Jak2 in the regulation of immune responses, we have limited our current study to APCs, particularly, to DC-mediated innate immune response. The implication of Jak2 in the regulation of adaptive immunity is yet to be determined. We are also aware that Jak2 is essential for the development and functionality of other immune cells such as T and B cells. Future studies focused on demonstrating these questions would shed light to the development of novel therapeutic approaches to diseases such as cancer and autoimmune disorders [52].

In conclusion, we have demonstrated the function of Jak2 in the regulation of DC development, maturation and cytokine secretion. Our data strongly support that Jak2 only selectively regulates the capacity of DCs to initiate innate immune response, while their capacity to stimulate T cell activation is not impacted. As a result, mice deficient for Jak2 show a DC-dependent resistance to lethal dose of LPS-induced septic shock, a deadly disorder caused by the excessive innate immune response. Together, our data suggest that Jak2 could be a unique therapeutic target for the intervention and treatment of clinical septic shock.

## Materials and Methods

### Mice

*Stat4^−/−^* (*H-2^d^*), *Stat6^−/−^* (*H-2^d^*) and BALB/cJ (*H-2^d^*) mice were purchased from the Jackson's Laboratory. Cre-ERT2 transgenic mice under the control of human ubiquitin C promoter (*H-2^b^*), *Jak2^fl/fl^* mice (*H-2^b^*), C57/BL6 (*H-2^b^*) mice, Stat5b transgenic (Stat5-Tg) mice (*H-2^d^*) and OT-1 transgenic mice (*H-2^b^*) were bred and housed in the SPF facility of Medical College of Georgia (MCG). All studies were carried out in compliance with MCG and Tongji Medical College Animal Care and Use Committee guidelines.

### Induction of Jak2 Deficiency

*Cre^+/+^Jak2^fl/fl^* mice were intraperitoneally (i.p.) injected with tamoxifen (25 mg/kg body weight) for 5 consecutive days. Tamoxifen was freshly dissolved in corn oil supplemented with 10% ethanol before injection. *Cre^+/+^Jak2^fl/fl^* littermates injected with equal volume of corn oil with 10% ethanol were served as controls.

### Generation of BMDCs

Bone marrow cells were flushed from femurs and tibias, and 5×10^5^/ml cells were plated in 150-mm Petri dishes and cultured with RPMI-1640 supplemented with 10% FCS, GM-CSF (10 ng/ml) and IL-4 (5 ng/ml) (PeproTech, Rocky Hill, NJ). Suspended cells were discarded at day-4 and the cultures were replaced with fresh media at day-7. BMDCs were then stimulated with LPS (0.5 µg/ml, Sigma, St Louis, MO) overnight and harvested on day-10 for experimental purpose [53].

### Isolation of PEM

PEM were collected by peritoneal lavage as reported with minor modifications [54]. Briefly, mice were i.p. injected with 5 ml sterilized cold RPMI 1640. PEM were harvested by washing peritoneal lavage twice with 5 ml cold RPMI 1640. After lysis of red blood cells, the cells were incubated for 3 h at 37°C in 35×15 mm culture dishes. Non-adherent cells were removed by exhaustive washing with 1 x PBS and the adherent cells were collected for experimental purpose.

### Western Blot Analysis

Total proteins were prepared from BMDCs or splenocytes using RIPA lysis buffer supplemented with protease inhibitors. Western blot analysis was carried out as reported by probing the blots with an indicated primary Ab (Santa Cruz, CA) followed by an HRP-conjugated secondary antibody. The reactive bands were visualized using an ECL Plus™ Western blot kit (PIERCE, Rockford, IL) [55]. β-actin was used for normalization. All Western blotting antibodies were purchased from Santa Cruz.

### Flow Cytometry Analysis

BMDCs or splenic single cell suspensions were prepared and washed with PBS. After blocking with anti-mouse CD16/32 Fcγ III/II receptor, the cells were stained for CD11c, CD11b, I-A^d^, CD80, CD86, and CD54 as reported [56]. After washes, the cells were suspended in FACS buffer and analyzed on a FACSCalibur (BD Bioscience, San Jose, CA). The data were analyzed using CellQuest v3.3 software as instructed. All flow cytometry antibodies were purchased from BD Bioscience.

### ELISA Analysis of Cytokines

The amount of TNFα, IL-2, IL-10, IL-12, IL-17 and IFN-γ in the culture media and sera was determined using the sandwich ELISA kits (eBioscience, San Diego, CA) as reported [57].

### Mixed Lymphocyte Reaction (MLR)

Splenic DCs originated from *Jak2^−/−^* and control mice were purified using a mouse DC enrichment kit (StemCell, Seattle, WA). For allogenic MLR, T cells isolated from BALB/cJ mice were co-cultured with irradiated (2000 rad) BMDCs or splenic DCs (2×10^5^/ml, DC∶T = 1∶10) originated from *Jak2^−/−^* or control mice for 56 h followed by additional 16 h in the presence of 0.5 µCi/well ^3^H thymidine. For antigen-specific MLR, 2.5×10^5^/ml irradiated (2000 rad) *Jak2^−/−^* BMDCs or splenic DCs were pulsed with 1 µM OVA peptide and then co-cultured with 5×10^5^/ml splenocytes isolated from OT-1 transgenic mice as above. After washes, the cells were finally harvested on glass wool filters with a suction-water-wash apparatus, dried, and counted in a beta scintillation counter [57]. T cell proliferation was estimated as mean CPM (counts per minute) ± SE.

### *Listeria monocytogenes* Infection

*Jak2^−/−^* and control mice were i.v. injected with 1×10^6^ CFU (∼1 LD_50_) *Listeria Monocytogenes* (LM-ova, a modified *Listeria* strain expressing OVA) in PBS as reported [58], [59]. Determination of *Listeria Monocytogenes* titer was carried out as previously described with minor modifications [59]. Briefly, spleens and livers were harvested after 48 h infection and homogenized in sterile distilled water containing 0.2% triton X-100. After 30 min incubation at room temperature, serial 10-fold dilutions were plated on TSB-agar and incubated at 37°C. Colonies were counted after 48 h incubation.

### *Stat3* siRNA Transfection in DC2.4 Cells

DC2.4 cells were transfected with a *Stat3* siRNA or a scramble RNA (Santa Cruz, CA) as instructed by the manufacturer. The cells were stimulated with 0.5 µg/ml LPS after 24 h transfection. Cell lysates and culture supernatants were then subjected to Western blot and ELISA analysis after 24 h stimulation, respectively.

### Induction of LPS-Challenged Septic Shock

For induction of septic shock, the mice were first i.p. injected with 50 mg/kg body weight of LPS (Sigma, St Louis, MO), and then subjected to evaluation of mortality and behavior changes every 6 h for >96 h. Mice injected with 150 µg LPS were used as a model for nonlethal sepsis. Serum samples were collected using whole blood after 12 h of injection. For adoptive transfer studies, both *Jak2^−/−^* and control mice were first underwent 800 rads of irradiation. Two days later, 1×10^7^ BMDCs derived from nonirradiated control mice were injected into these irradiated mice via tail vein, respectively. After 4 h of transfer, the mice were underwent induction of septic shock with lethal dose of LPS (25 mg/kg body weight) as above. Eight mice were included in each study group.

### Statistical Analysis

Survival curves for septic shock were generated by the Kaplan and Meier method. *Chi-square test* was employed to determine the difference of mortality for LPS-induced septic shock. Comparisons between groups for flow cytometry, cytokine and MLR data were accomplished by one-way ANOVA using SPS 11.5 for windows. Data were present as mean ± SE. *P*<0.05 was considered statistically significant.

## Supporting Information

Figure S1 Loss of Jak2 suppresses the maturation of splenic DCs. Surface marker expressions were analyzed by flow cytometry in splenic DCs. Each bar represents the average percentage of DCs positive for the surface marker analyzed.
(0.12 MB TIF)

Figure S2 AG490 suppresses BMDC maturation in a dose-dependent manner. Bone marrow cells derived from B6 mice were used to generate BMDCs. AG490 (0, 100 and 200 µM, respectively) was added into the cultures on day-4. BMDCs were stimulated with LPS (500 ng/ml) on day-9 and harvested on day-10 for flow cytometry analysis of surface marker expressions. Left panel: a representative flow cytometry data of three independent experiments performed; right panel: a bar graphic figure showing the average percentage of BMDCs positive for each surface marker analyzed. *, p<0.05.
(4.21 MB TIF)

Figure S3 Tamoxifen does not have perceptible effect on DC phenotype. 25 mg/kg body weight of tamoxifen or carrier solution were i.p. injected into B6 mice for 5 consecutive days. The mice were sacrificed after 2 wk of last injection. Bone marrow cells were used to generate BMDCs and surface marker expressions were analyzed before/after LPS stimulation.
(0.67 MB TIF)

Figure S4 Loss of Jak2 suppresses surface marker expressions in macrophages. Macrophages deficient for Jak2 show less matured phenotype. Each bar represents the average percentage of macrophages positive for each surface marker analyzed.
(0.13 MB TIF)

Figure S5 IL-10 secretion after LPS challenge (A) ELISA analysis of IL-10 production of culture supernatants derived from Jak2−/− and wild-type BMDCs before/after LPS (1 µg/ml) treatment. (B) Serum IL-10 levels after LPS challenge. Both Jak2−/− and control mice were first challenged with nonlethal dose of LPS (150 µg/mouse) and 12 h later the mice were sacrificed. Sera were pooled from three mice and then subjected to ELISA analysis of IL-10.
(0.12 MB TIF)

Figure S6 Flow cytometry analysis of apoptotic BMDCs and splenic DCs after LPS challenge (A) BMDC apoptosis after LPS stimulation. BMDCs were stimulated 10 µg/ml LPS for 96 h and then stained with Annexin-V and PI followed by flow cytometry analysis of apoptotic cells. (B) Splenic DC apoptosis after LPS challenge. Jak2−/− and control mice were first challenged with 150 µg/mouse of LPS and were sacrificed after 20 h of injection. Splenic DC apoptosis was then analyzed by flow cytometry as above.
(0.11 MB TIF)

Figure S7 Western blot analysis of the activation states of Jak2 downstream molecules in macrophages. Only STAT3 and STAT4 showed defective activation in Jak2−/− macrophages.
(0.16 MB TIF)
