# Supplementary figures and images for: Loss of Jak2 Selectively Suppresses DC-Mediated Innate Immune Response and Protects Mice from Lethal Dose of LPS-Induced Septic Shock
Source: PLoS One. 2010 Mar 9;5(3):e9593. doi: 10.1371/journal.pone.0009593 (PMC2834745; doi:10.1371/journal.pone.0009593)

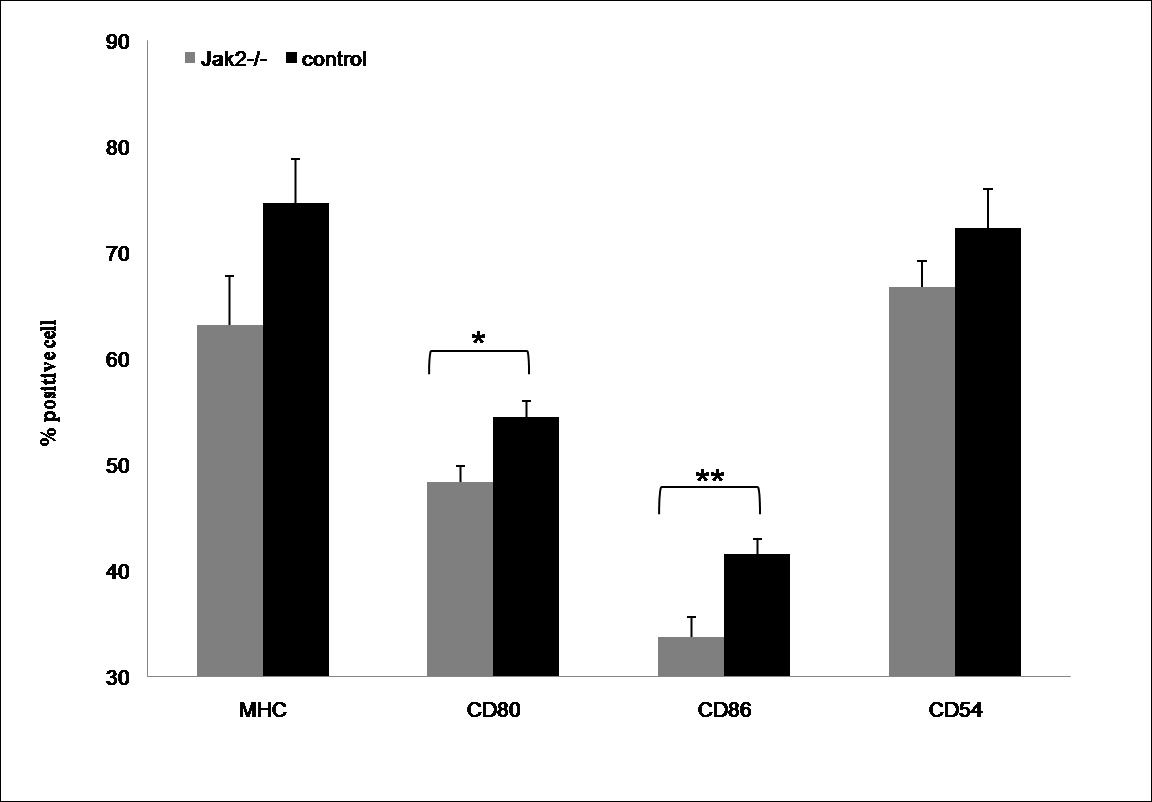

Supplement: Figure S1 — Loss of Jak2 suppresses the maturation of splenic DCs. Surface marker expressions were analyzed by flow cytometry in splenic DCs. Each bar represents the average percentage of DCs positive for the surface marker analyzed. (0.12 MB TIF) [file pone.0009593.s001.tif]

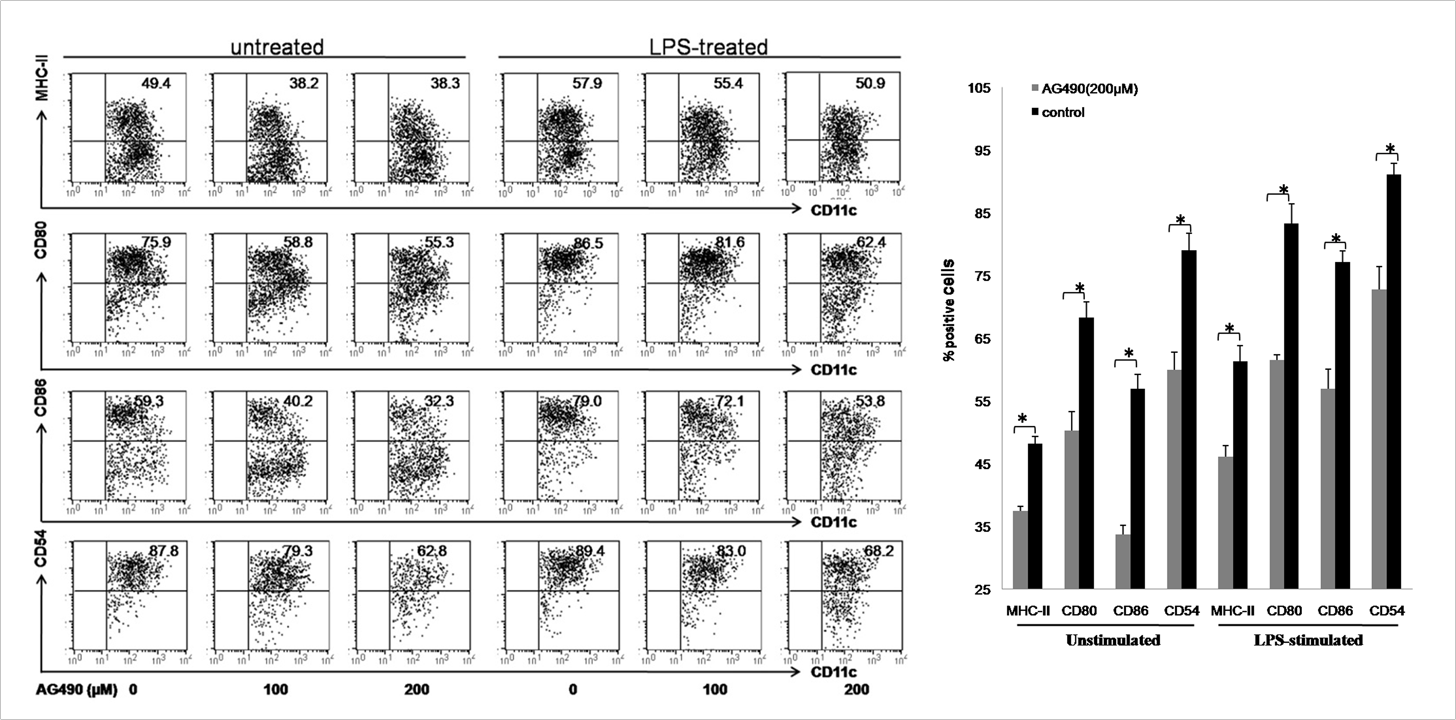

Supplement: Figure S2 — AG490 suppresses BMDC maturation in a dose-dependent manner. Bone marrow cells derived from B6 mice were used to generate BMDCs. AG490 (0, 100 and 200 µM, respectively) was added into the cultures on day-4. BMDCs were stimulated with LPS (500 ng/ml) on day-9 and harvested on day-10 for flow cytometry analysis of surface marker expressions. Left panel: a representative flow cytometry data of three independent experiments performed; right panel: a bar graphic figure showing the average percentage of BMDCs positive for each surface marker analyzed. *, p<0.05. (4.21 MB TIF) [file pone.0009593.s002.tif]

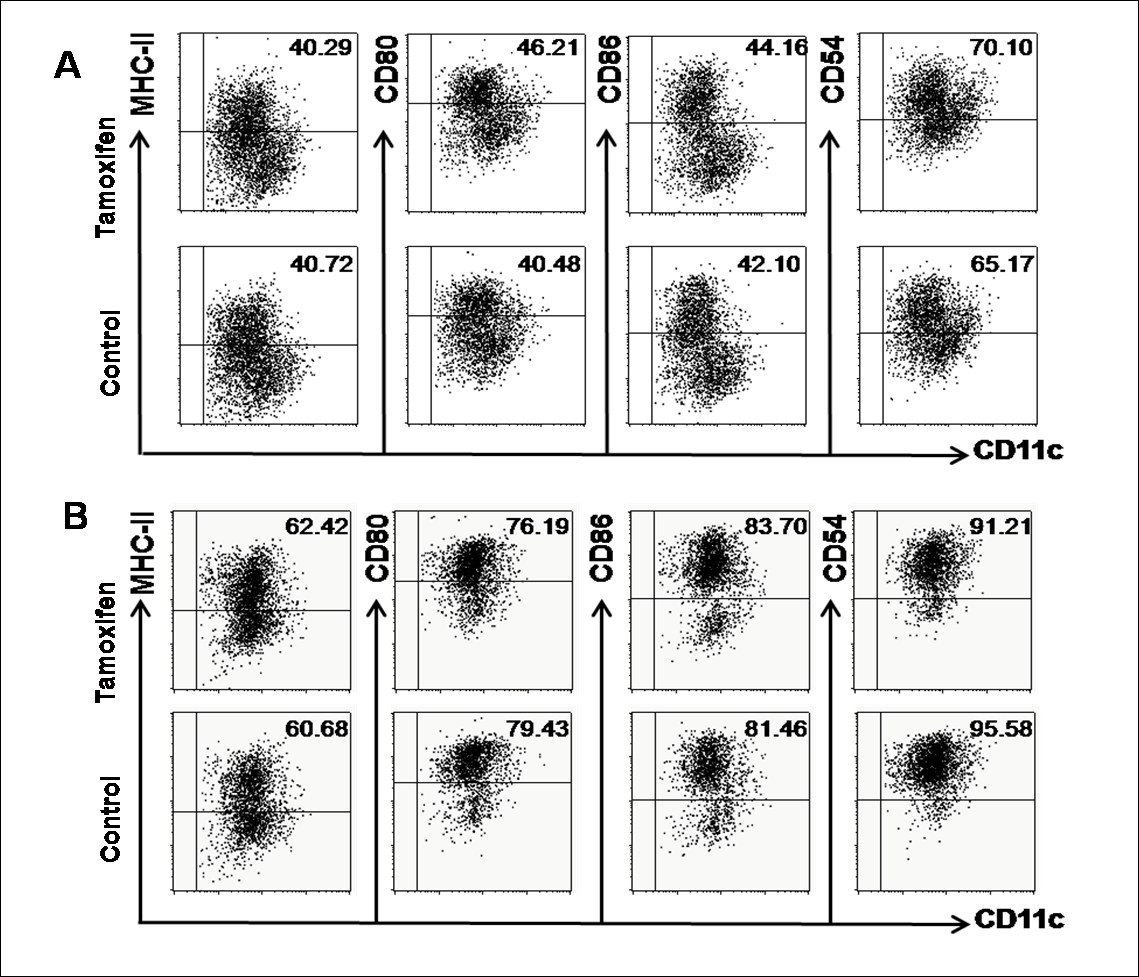

Supplement: Figure S3 — Tamoxifen does not have perceptible effect on DC phenotype. 25 mg/kg body weight of tamoxifen or carrier solution were i.p. injected into B6 mice for 5 consecutive days. The mice were sacrificed after 2 wk of last injection. Bone marrow cells were used to generate BMDCs and surface marker expressions were analyzed before/after LPS stimulation. (0.67 MB TIF) [file pone.0009593.s003.tif]

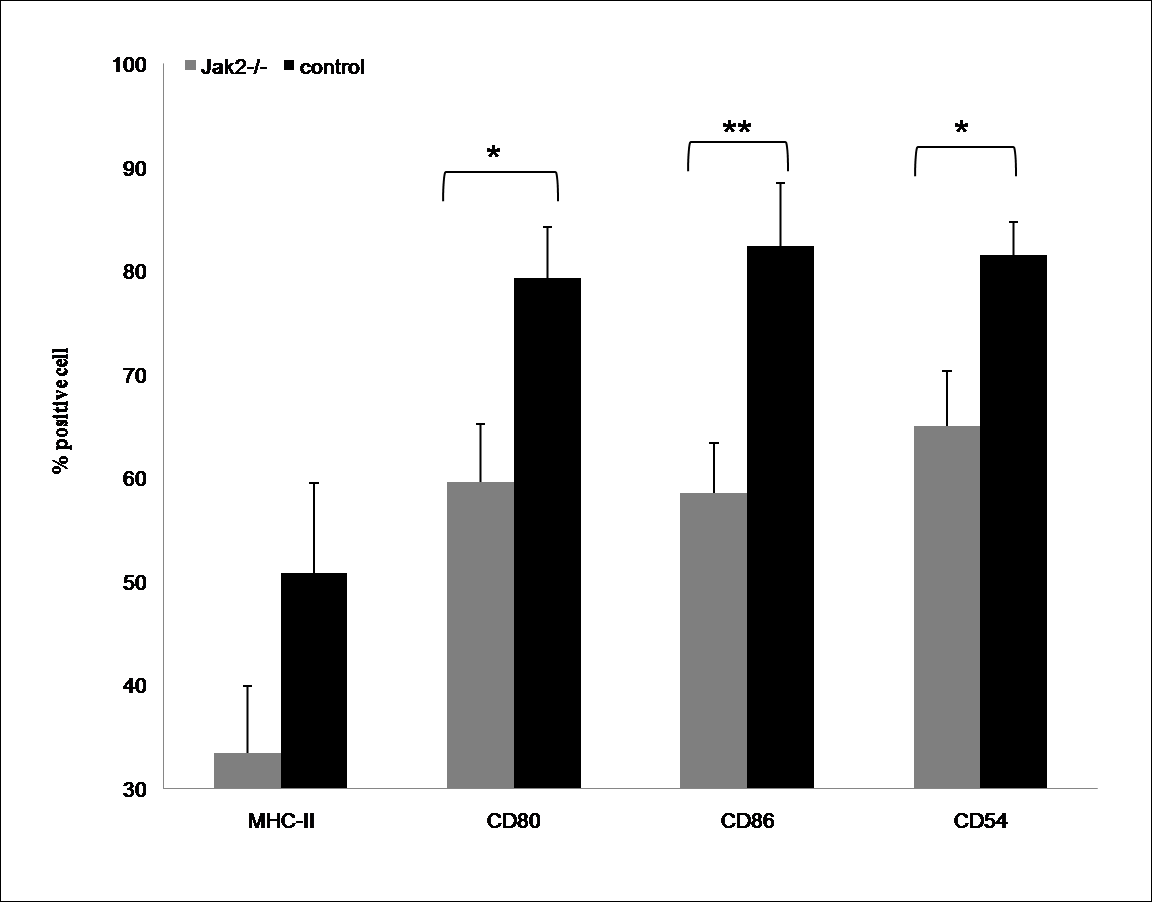

Supplement: Figure S4 — Loss of Jak2 suppresses surface marker expressions in macrophages. Macrophages deficient for Jak2 show less matured phenotype. Each bar represents the average percentage of macrophages positive for each surface marker analyzed. (0.13 MB TIF) [file pone.0009593.s004.tif]

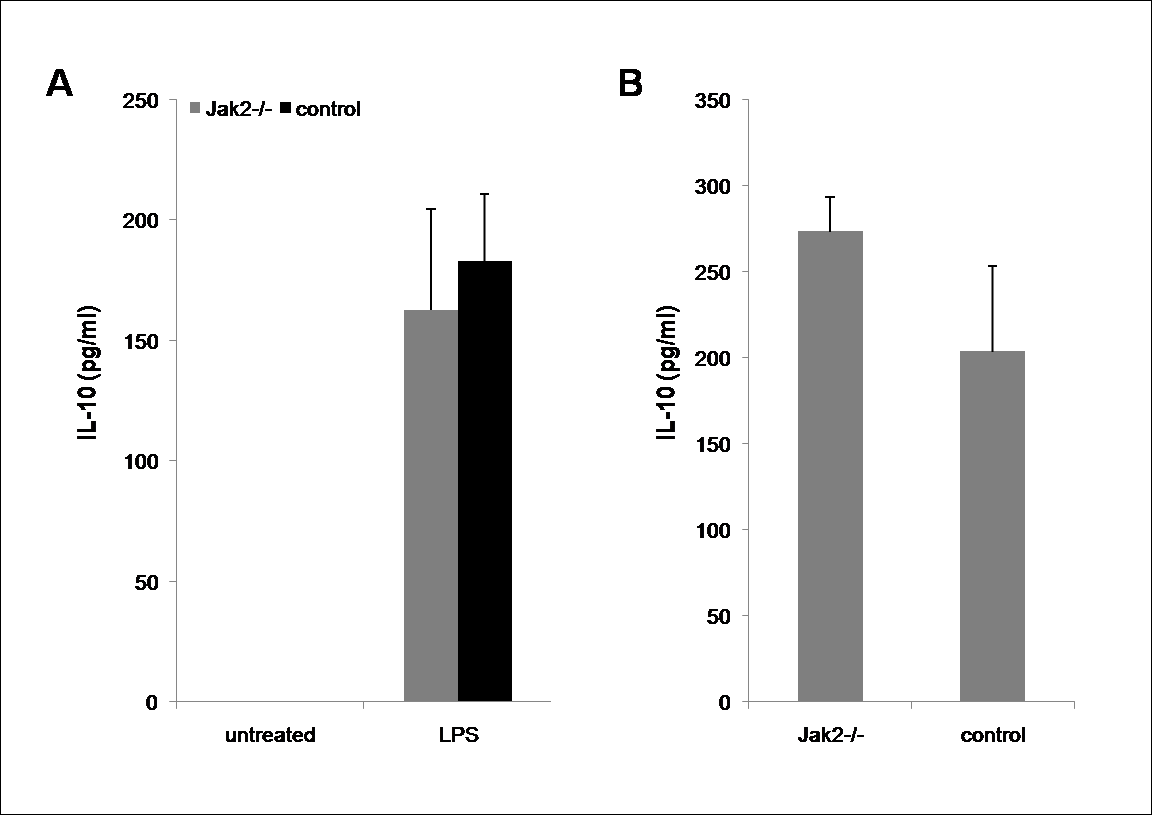

Supplement: Figure S5 — IL-10 secretion after LPS challenge (A) ELISA analysis of IL-10 production of culture supernatants derived from Jak2−/− and wild-type BMDCs before/after LPS (1 µg/ml) treatment. (B) Serum IL-10 levels after LPS challenge. Both Jak2−/− and control mice were first challenged with nonlethal dose of LPS (150 µg/mouse) and 12 h later the mice were sacrificed. Sera were pooled from three mice and then subjected to ELISA analysis of IL-10. (0.12 MB TIF) [file pone.0009593.s005.tif]

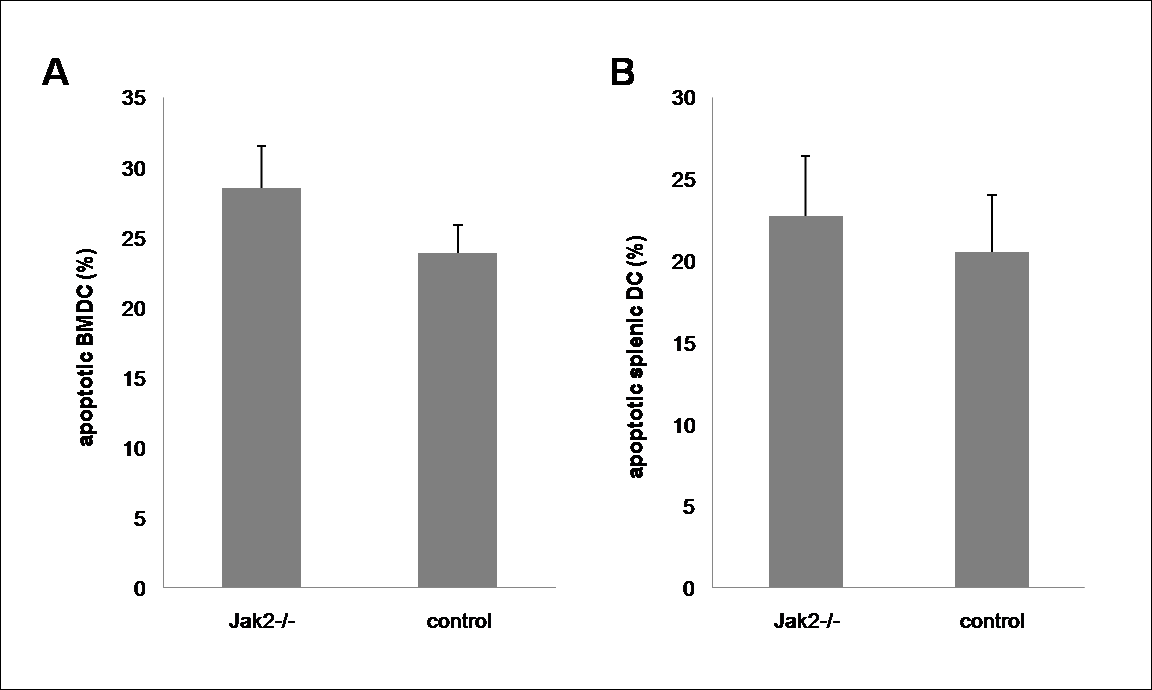

Supplement: Figure S6 — Flow cytometry analysis of apoptotic BMDCs and splenic DCs after LPS challenge (A) BMDC apoptosis after LPS stimulation. BMDCs were stimulated 10 µg/ml LPS for 96 h and then stained with Annexin-V and PI followed by flow cytometry analysis of apoptotic cells. (B) Splenic DC apoptosis after LPS challenge. Jak2−/− and control mice were first challenged with 150 µg/mouse of LPS and were sacrificed after 20 h of injection. Splenic DC apoptosis was then analyzed by flow cytometry as above. (0.11 MB TIF) [file pone.0009593.s006.tif]

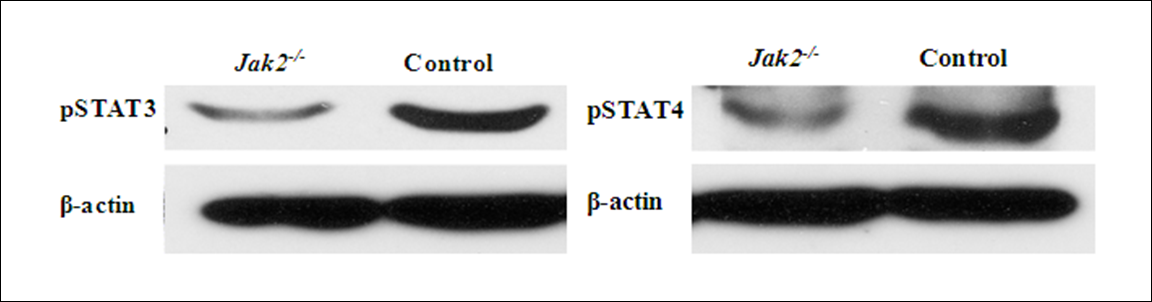

Supplement: Figure S7 — Western blot analysis of the activation states of Jak2 downstream molecules in macrophages. Only STAT3 and STAT4 showed defective activation in Jak2−/− macrophages. (0.16 MB TIF) [file pone.0009593.s007.tif]
